# Mechanical Properties of Longitudinal Basalt/Woven-Glass-Fiber-reinforced Unsaturated Polyester-Resin Hybrid Composites

**DOI:** 10.3390/polym12102211

**Published:** 2020-09-27

**Authors:** S.M. Sapuan, H.S. Aulia, R.A. Ilyas, A. Atiqah, T.T. Dele-Afolabi, M.N. Nurazzi, A.B.M. Supian, M.S.N. Atikah

**Affiliations:** 1 Advanced Engineering Materials and Composites Research Centre (AEMC), Department of Mechanical and Manufacturing Engineering, Faculty of Engineering, Universiti Putra Malaysia, UPM Serdang 43400, Selangor, Malaysia; sarahylaea@gmail.com (H.S.A.); deleafolabitemitope@gmail.com (T.T.D.-A.); mohdsupian7779@gmail.com (A.B.M.S.); 2 Laboratory of Technology Biocomposite (BIOCOMPOSITE), Institute of Tropical Forestry and Forest Products (INTROP), Universiti Putra Malaysia, UPM Serdang 43400, Selangor, Malaysia; 3 Institute of Microengineering and Nanoelectronics, Level 4, Research Complex, Universiti Kebangsaan Malaysia, Bangi 43600, Malaysia; atiqahafdzaluddin@gmail.com; 4 Centre for Defence Foundation Studies, National Defence University of Malaysia, Kem Sungai Besi, Kuala Lumpur 57000, Malaysia; mohd.nurazzi@gmail.com; 5 Department of Chemical and Environmental Engineering, Faculty of Engineering, Universiti Putra Malaysia, UPM Serdang 43400, Selangor, Malaysia; sitinuratikah_asper7@yahoo.com

**Keywords:** basalt fiber, glass fiber, natural-fiber composites, hybrid composites, mechanical properties

## Abstract

This work represents a study to investigate the mechanical properties of longitudinal basalt/woven-glass-fiber-reinforced unsaturated polyester-resin hybrid composites. The hybridization of basalt and glass fiber enhanced the mechanical properties of hybrid composites. The unsaturated polyester resin (UP), basalt (B) and glass fibers (GF) were fabricated using the hand lay-up method in six formulations (UP, GF, B7.5/G22.5, B15/G15, B22.5/G7.5 and B) to produce the composites, respectively. This study showed that the addition of basalt to glass-fiber-reinforced unsaturated polyester resin increased its density, tensile and flexural properties. The tensile strength of the B22.5/G7.5 hybrid composites increased by 213.92 MPa compared to neat UP, which was 8.14 MPa. Scanning electron microscopy analysis was used to observe the fracture mode and fiber pullout of the hybrid composites.

## 1. Introduction

A composite material is a nonuniform solid produced by combining two or more materials that are mechanically bonded together. Each material in a composite retains its properties, and when combined, their combined properties improves their properties as individual solids [1,2,3,4,5]. In general, composites are composed of two phases, the matrix and the reinforcement. The matrix serves to bond the reinforcements, which in turn, increase the strength of the composite [4,6,7]. Today, composites composed of fiber and polymer are the most popular and are widely applied in various types of industries [8,9,10,11,12]. They have the potential to substitute for conventional metals in structural applications like aerospace, automobile and in wind-turbine-blade manufacturing [12,13,14]. The most well-known, fiber-reinforced polyester composites in which continuous fibers material embedded in a polyester matrix are combination of natural-fiber-reinforced polyester (NFRP), glass-fiber-reinforced polyester (GFRP) and carbon-fiber-reinforced polyester (CFRP) [15,16,17,18,19].

Basalt originates from volcanic magma and flood volcanoes. It is a fluid or semifluid material that is extremely high in temperature beneath the earth crust and solidifies in open air [20,21,22,23,24]. Basalt rocks are formed from molten lava after solidification, thus categorized as volcanic rocks. The appearance of basalt rocks is dark colored or grayish [25]. Basalt fibers are considered to be fibers that are of high strength, have excellent modulus properties, high-temperature resistance, easy to fabricate, inexpensive, nontoxic, natural and eco-friendly [26]. Therefore, basalt fibers have recently been explored in many studies to further investigate their mechanical, physical, chemical and thermal properties, as well as the enhancement of its strength and the application for industries [27,28,29].

Glass fiber is one of the prominent materials that is applied in numerous applications such as building automobile bodies, thermal and electrical insulations, various sports goods, household goods and many industrial applications [30]. However, when it comes to high-strength applications, higher-strength carbon and Kevlar fibers offer better performance compared to glass fiber [31,32]. From the perspective of production cost, glass fiber is lower in price compared to carbon fibers. Therefore, to maintain a low cost of fiber-reinforced materials industrials applications, it is essential to combine various fibers with glass fibers to form a hybrid composite with new properties that are suitable for various applications. In addition, glass fiber is a strong and tough material, therefore, hybridization of glass fiber will bring advantages in terms of cost and strength of the materials [32,33].

Hybrid composites are those composites that are formed by combination of two or more various types of fibers as reinforcement in a matrix [34,35]. Hybrid composites offer characteristics and properties that cannot be obtained with single-fiber reinforcement. Hybridization allows producers to modify composite properties to the exact requirements of a different structure consideration. The objective of hybridization is to produce a new material that maintain the advantages of its constituents at lower cost. However, some fiber reinforcements in hybrid composite could be more expensive than others [36]. The same paper also mentioned that hybrid composites are classified as interply and intraply hybrid composites. In interply hybrid composites, layers of two or more homogeneous fibers are stacked in layer with various stacking sequences while in intraply hybrid composites, the homogeneous fibers are mixed in the same layer. Towards preserving human health and environment, more researchers and scientists studied the incorporation of natural fiber, such as kenaf [37,38], flax [39], jute [40,41] and sugar palm [42,43] with glass fiber in hybrid composites.

Berozashvili et al. [44] stated that basalt fiber is a novel fiber appearing in recent years. It offers high strength, excellent fiber or resin adhesion and ability to be easily processed using conventional processes and equipment. Furthermore, basalt fibers are free from any other additives in a single production process, which is an advantage in production cost even compared to glass fiber. Compared to e-glass fiber, basalt fibers have higher tensile strength. In terms of strain to failure, basalt fibers has larger failure compared to carbon fibers. In addition, basalt fibers have high chemical stability, great mechanical properties, good noise dampening properties, perfect thermal resistance (superior to glass fibers), chemical resistant and low water- logging materials. On top of this, basalt fibers are nontoxic, noncombustible, bioinert and harmless to the human health [45,46]. According to Wei et al. [47], the basalt fibers are suitable for various applications, such as corrosion-resistant materials in the chemical industry, wear and friction materials in the automobile industry, target areas of anti-low velocity impact, construction reinforcement materials and high-temperature insulation of automobile catalysts.

The purpose of this research is to investigate the hybridization of glass fibers with other types of fibers that offer high strength—as well as being more environmentally friendly. Glass- and basalt-fiber hybridizations are still less popular than hybridization with glass fiber with other synthetic or natural fiber. The classification of basalt fiber is actually debatable. Some studies mentioned that basalt fiber is natural fiber from mineral source, since it does not require any additive material during fabrication [22].

## 2. Experimental

### 2.1. Materials

The materials used during this study were unsaturated polyester resin (UP), methyl ethyl ketone peroxide (MEKP) as the curing catalyst, woven glass fiber (GF) and roving basalt fiber (B). The woven glass fiber was supplied by Innovative Pultrusion Sdn. Bhd. (Negeri Sembilan, Malaysia) and the roving basalt fiber was supplied by Basaltex NV (Wevelgem, Belgium). The woven glass fiber was cut into 300 mm × 300 mm pieces for each layer. The basalt fiber was cut into 300-mm-long pieces for each fiber and was randomly distributed in each layer during the fabrication.

### 2.2. Fabrication of B/GF/UP Hybrid Composites

The fabrication of the composites was by hand lay-up technique in a steel mold with dimension (*l* × *w* × *t*) 300 mm × 300 mm × 5 mm, then were compressed using compression molding (40 tons). The fibers and the unsaturated polyester resin were fabricated into composites as sandwich-structured composite. All composites in this study consists of 30 wt % fiber and 70 wt % unsaturated polyester matrix, as shown in Table 1. For the hybrid composites, three different weight percentages (wt %) of glass–basalt fiber were fabricated to investigate the effect of various fiber compositions to the mechanical (tensile and flexural) properties of the hybrid composites.

### 2.3. Density

A densimeter (Mettler-Toledo (M) Sdn. Bhd, Selangor, Malaysia) was used in the determination of the density of acquired B/G/UP hybrid composites. The samples were dried for 7 days in desiccators with P_2_O_5_ as the drying agent. Afterward, the computation of the initial dry matter of each hybrid composites was accomplished. The hybrid composites samples were weighed (*m*) before immersing hybrid composites into the liquid of volume (*V*) and the density denoted as (*ρ*), was calculated from Equation (1). Each test was carried out 6 times.

(1) $$\rho =\frac{m}{V}$$

### 2.4. Tensile Test

A tensile test was performed to measure the force required to stretched and elongated the composite until it reached the break point. Tensile test was performed at specimen temperature of 23 °C and relative humidity of 50%, according to the standard ASTM D3039 using INSTRON 3365 (INSTRON, University Ave, Norwood, MA, US) test machine. Each sample dimension (*l* × *w* × *t*) was 120 mm × 20 mm × 5 mm. The gauge length was 60 mm and the strain rate was set at 5 mm/min.

### 2.5. Flexural Test

A flexural test was performed according to standard ASTM D790 [48] that employ three-point loading system applied to a simply supported beam with the strain limit of the test at 5.0%. The span to depth ratio was 16:1 and the crosshead speed was 5 mm/min. Each sample’s dimensions (*l* × *w* × *t*) were 127 mm × 13 mm × 5 mm.

### 2.6. Scanning Electron Microscopy (SEM)

The post tensile testing of B/G/UP hybrid composites was observed using an EM-30AX scanning electron microscope (SEM; COXEM, Daejon, Korea) with an acceleration voltage of 20 kV. All six formulations of B/G/UP hybrid composites were coated with a thin layer of gold prior to morphology analysis.

### 2.7. Statistical Analysis

SPSS software was used to perform the analysis of variance (ANOVA) on the obtained experimental results. Tukey’s test was employed to conduct means comparisons at a 0.05 level of significance (*p* ≤ 0.05).

## 3. Result and Discussion

### 3.1. Density

**Figure 1:**
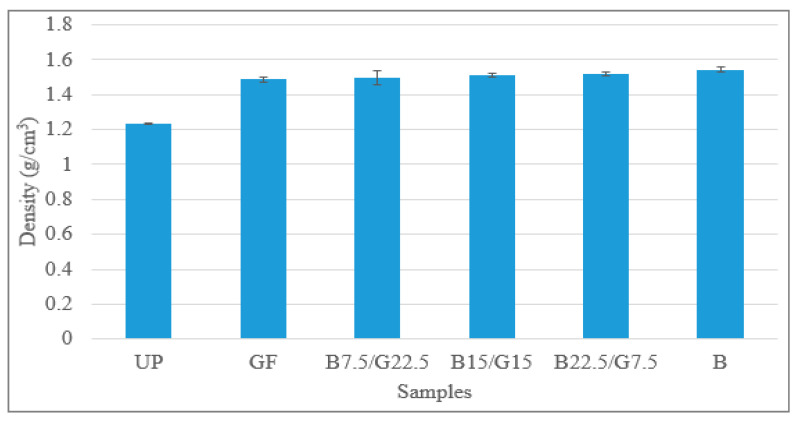
Density of B/G/UP hybrid composites.

The density of UP, GF, B, B7.5/G22.5, B15/G15 and B22.5/G7.5 hybrid composites are shown in Figure 1. The B composites had higher density than B22.5/G7.5 hybrid composites, which were 1.54 g/cm^3^ and 1.51 g/cm^3^, respectively. As the basalt content increased from 7.5 to 22.5 wt %, the density also increased. The density of B22.5/G7.5 hybrid composites was B7.5, compared to B15/G15 and B7.5/G22.5. Nevertheless, the B composite had a higher density than the G and UP composites. Referring to Nurazzi et al. [49], the experimental density of composites decreased as the sugar palm fiber loading increase [49]. This may have been due to the lower density of sugar palm fibers (1.053 g/cm^3^) compared to the polyester resin (1.212 g/cm^3^). However, in this study, the main reason of the high density of B composite is the density of basalt fiber [50] that is higher than both glass fiber and unsaturated polyester [51].

### 3.2. Tensile Properties

The presence of basalt fiber in the hybrid composite with glass fiber would enhance the tensile properties because basalt fiber has higher strain, strength and modulus than glass fiber [52]. The highest tensile strength was observed for B22.5/G7.5, which was 269.85 ± 23.35 MPa, followed by B, B15/G15, B7.5/G22.5, G and UP, which were 213.92 ± 5.31, 168.89 ± 11.62, 147.69 ± 15.27, 126.33 ± 16.43 and 8.14 ± 1.23 MPa, respectively. Therefore, the increment of basalt fiber (wt %) enhances the tensile properties. Similar results were also found in the previous works that the replacement of glass mat to basalt fiber resulted in enhanced mechanical property of GFRP [53]. However, according to Amuthakkannan et al. [54], a similar result was also found. The glass–basalt hybrid composite with 8B/4G had better tensile properties compared to BFRP, which indicated that the presence of glass fiber (even below the percentage of basalt fiber) would improve the tensile properties of the composite.

For the composite consisting only one type of fiber, sample B achieved higher strength compared to sample G. The average tensile strength of composite B was 213.92 MPa and sample G was 126.33 MPa. Therefore, based on the results, BFRP had higher tensile strength compared to GFRP.

**Figure 2:**
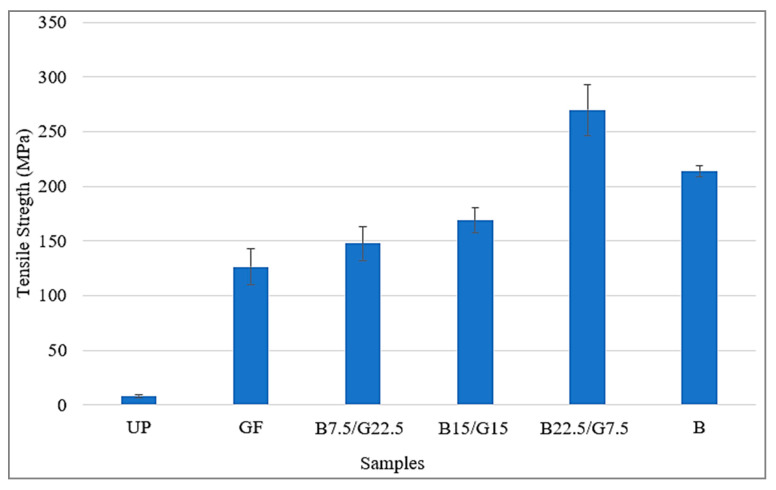
Tensile strength of basalt/glass hybrid composites.

Figure 2 shows the trend of the tensile strength results. It can be seen that the basalt fiber added to hybrid composite was able to increase the tensile strength of the sample B7.5/G22.5 and B15/G15 compared to sample G. However, the tensile strength of the two composites were still lower compared to sample B that consisted of 30 wt % basalt fiber and 70 wt % unsaturated polyester resin. The best fiber percentage composition of glass–basalt fiber in hybrid composite was 75% basalt fiber and 25% glass fiber (sample B22.5/G7.5). Since the tensile strength of sample B22.5/G7.5 was the highest, it proved that in the percentage of glass/basalt fiber composition at 25%/75%, the hybrid composite was able to increase the maximum load at tensile test compared to GFRP and BFRP. In addition, the improvement of tensile strength of the composites can be attributed to the fact that an effective layering design of balance (B22.5/G7.5) was formed between the outer layer of the skin of the composite glass fiber mat and long and continuous of basalt fiber acted as a core and tendon for the composite structure. An effective layering and structural design of the composites resisted sudden and fast crack growth because microcrack propagation towards the core of the composites was stopped by the individual glass fiber mat at the outer surface. Moreover, an optimized matrix acted as a binder of the fibers, transferring the load to the fibers and providing rigidity and shape to the structure. This contributed to the improvement of mechanical properties of the composites [55].

**Figure 3:**
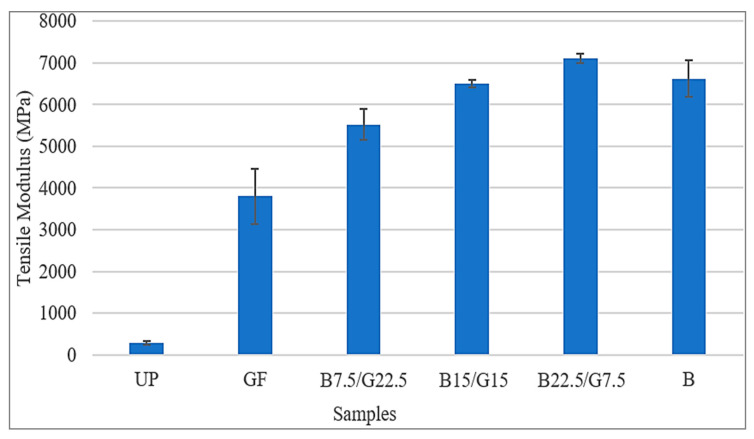
Tensile modulus of B/G/UP hybrid composites.

There is a correlation between tensile modulus and the tensile strength of the sample composites. A higher tensile modulus indicated higher elasticity of a material. From the results obtained, sample B22.5/7.5 achieved the highest tensile at average of 7106.32 MPa, followed by sample B at average of 6620.11 MPa, sample B15/G15 at average of 6503.5 MPa, sample B7.5/G22.5 and the lowest tensile modulus obtained by sample G at average of 3802.17 MPa. Figure 3 shows the trend of tensile modulus average values for each sample. It can be seen for the hybrid composites, the higher the weight percentage of basalt fiber, the higher the tensile modulus. However, from the results obtained in this study, sample B that consisted the highest weight percentage of basalt fiber possessed lower tensile modulus compared to sample B22.5/G7.5. A balance ratio between the basalt fiber and glass fiber is needed in providing the stiffness and toughness to the composite structure. This is because, the glass fiber’s outer layer performed as a skin to protect fast micro or macro cracking initiation that supported by the core of the composites which contributed by basalt fiber.

**Figure 4:**
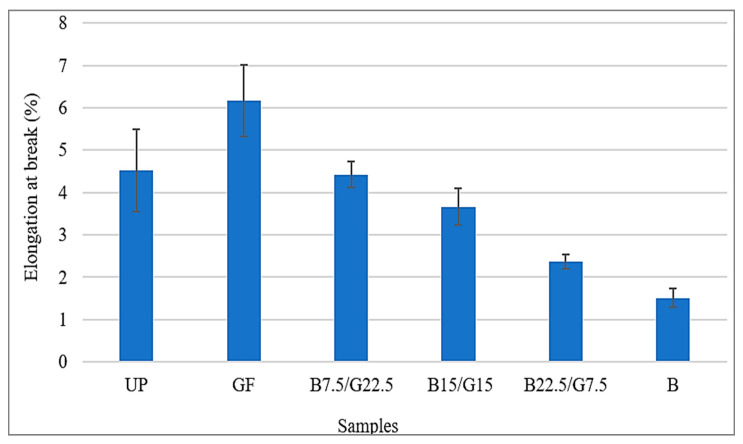
Elongation at break of B/G/UP hybrid composites.

Figure 4 shows the percentage elongation at break of the samples. Elongation at break is the ration between changed length after breakage and initial length of the material. Composite sample G that consisted of 30 wt % glass fiber showed the highest elongation and sample B that consisted of 30 wt % basalt fiber showed the lowest elongation at break. From the trend, it can be observed that the higher the basalt fiber contained in the composite, the lower the elongation at break. This is due to the improvement of rigidity of the composite after the improvement in the silica oxide (SiO_2_) and aluminum oxide (Al_2_O_3_) that had little contribution to the elongation but increased the modulus properties of the basalt fiber composite. Thus, basalt/glass fiber compared with glass fiber composite had a higher strain to failure characteristic compared to the low strain of extensibility of basalt fiber composite. The observed decrement in flexibility through decreasing elongation at break of the composites was likely related to the high stiffness of the composites with an increasing tensile modulus.

Table 2 presents the results of tensile properties of the samples. The results shown that composite B22.5/G7.5 achieved the highest tensile strength and the highest tensile modulus at average of 269.85 and 7106.32 Mpa. The lowest tensile strength and tensile modulus were achieved by sample G that consisted of 30 wt % of glass fiber and 70 wt % of unsaturated polyester resin. For the elongation at break, sample B had the lowest average value at 1.51% and sample G had the highest value at 6.17%.

Overall, based on the obtained results for hybrid composites, the higher the weight percentage of basalt fiber contained in the hybrid composite, the higher the strength of the tensile properties. However, BFRP had lower strength of tensile properties compared to B22.5/G7.5. Thus, the presence of glass fiber in glass–basalt reinforced hybrid composite with ratio fiber percentage of 25% glass fiber and 75% basalt fiber, will improve the overall tensile properties.

### 3.3. Scanning Electron Microscopy

**Figure 5:**
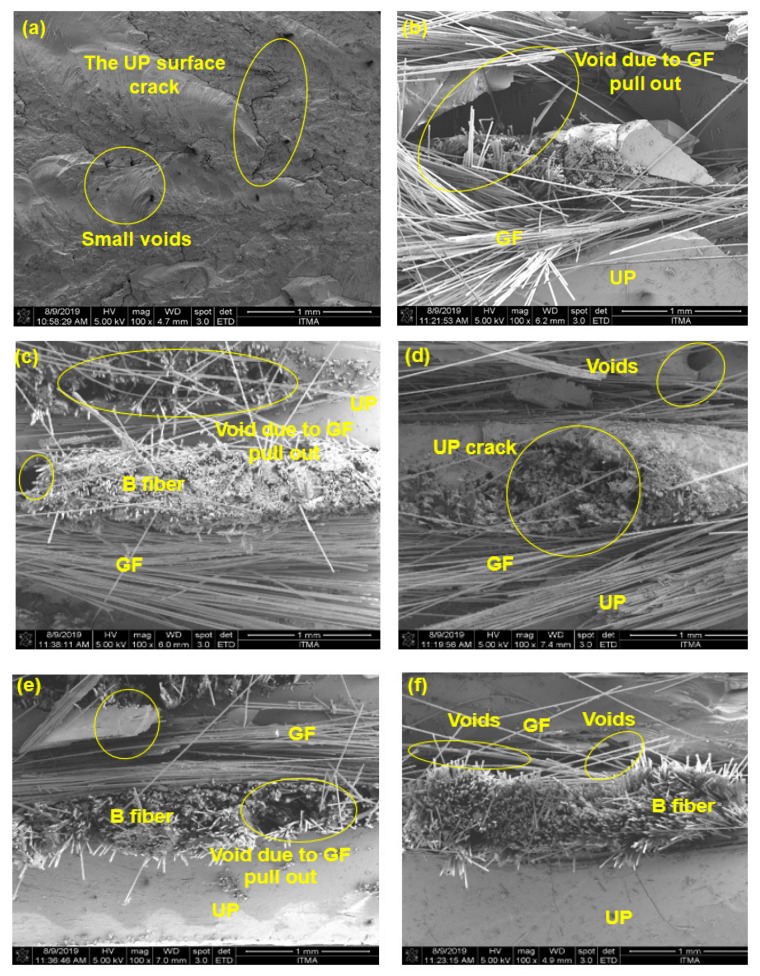
SEM morphology of (**a**) UP; (**b**) G; (**c**) B7.5/G22.5 (**d**) B15/G15; (**e**) B22.5/G7.5; (**f**) B post-tensile testing.

Figure 5a–f shows the morphology of basalt/glass-fiber-reinforced unsaturated polyester hybrid composites. For UP resin, there were microcracks appeared in the surface, as shown in Figure 5a. It was observed that more presence of fiber pull-out with increasing addition of basalt fiber even though initially, the UP surface was smooth than UP composites. The SEM morphology showed clear appearance of more fiber breakage and fewer voids as in Figure 5c–e. This was due to the debonding of the UP matrix when the samples were mechanically fractured, as in Figure 5e. This can be correlated in the tensile properties of B22.5/G7.5 that was improved than other composites. It was found that the B22.5/G7.5 hybrid composites in Figure 5e showed less matrix cracking and fiber debonding, which may be contributed to higher stress absorption resulting in higher tensile and flexural properties (Table 2 and Table 3). When the hybrid composites were in tension, the basalt fibers were able to resist the high tension, as well as to absorb a significant amount of tensile stress through the delamination of the glass fiber at the outer surface, fiber breakage and fiber pull-out of the glass fiber and induction of micro- to macro-cracking of the UP matrix [56,57,58].

### 3.4. Flexural Properties

According to previous study by Fiore et al. [53], the increase of basalt layer will improve the stiffness of the hybrid structures compared to the GFRP. Furthermore, during three-point bending test, the stress increment changed from the central zone to the external sides of the beam. The middle zone of the beam was in neutral axis since the top and bottom layer were subjected to the maximum stress. Therefore, since basalt fiber has better properties than glass fiber, the presence of basalt fiber in the hybrid composite enhance the flexural properties compared to GF composites. From the results obtained for GF and B composites, it was shown that basalt fiber reinforced polymer composite had higher flexural strength and Young’s modulus compared to sample G. The flexural strength of B composites was higher by 380% to the flexural strength of GF composites. Furthermore, young’s modulus of B composites was also higher by 250% compared to Young’s modulus of GF composites.

**Figure 6:**
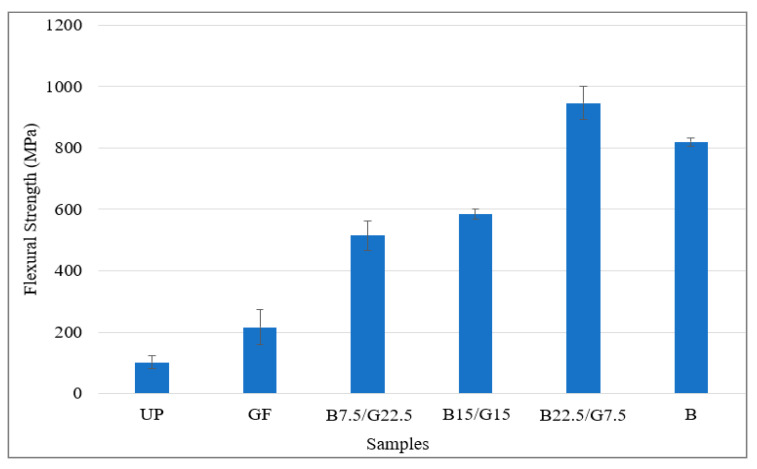
Flexural strength of B/G/UP hybrid composites.

**Figure 7:**
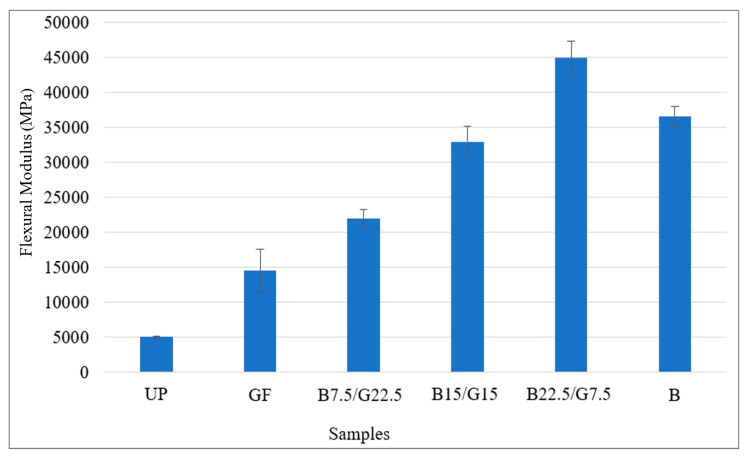
Young modulus of B/G/UP hybrid composites.

Figure 6 shown the trend of the flexural strength results. Figure 7 displays the trend of Young’s modulus results. For the hybrid composite, from the trend, the higher the basalt fiber contained in the composite, the higher the flexural strength and Young’s modulus. However, sample B22.5/G7.5 had a higher tensile strength and Young’s modulus compared to sample B, even though sample B contained higher weight percentage of basalt fiber.

As depicted in Figure 6 and Figure 7, the flexural strength and modulus of the hybrid composites have similar trends as the tensile properties. The flexural properties increased with the increase of basalt fiber. In flexural test, failures mainly occurred due to bending and shearing [59]. The increased flexural strength of the hybrid composites with the loading of basalt fiber was mainly due to the increased resistance to shearing of the composites. The rigid basalt fiber was effectively acted as a core for the composites and a further increase of basalt fiber content in the hybrid composites resulted in the composite having sufficient modulus [60]. This was due to the fibers which were present in a sufficient amount, providing an effective stress transfer between the matrix and fiber and also due to the inherent properties of basalt fiber in the middle. This suggested that the flexural properties of the composites were more dependent on the amount of basalt fiber rather than glass fiber, which may be due to the high modulus of the basalt fiber compared to glass fiber that contributed by their unique chemical compositions [61].

Table 3 shows the results of flexural properties of the composite samples. Overall, the sample B22.5/G7.5 had the average of flexural strength and Young’s modulus which were at 946.46 and 44,890.05 MPa, respectively. The lowest results for average flexural strength and Young’s modulus achieved by sample G were 215.72 and 14,546.90 MPa, respectively. Overall, based on the obtained results for hybrid composites, the higher the weight percentage of basalt fiber contained in the hybrid composite, the higher the flexural strength. However, BFRP had lower strength in flexural properties compared to B22.5/G7.5. Thus, the presence of glass fiber in glass–basalt reinforced hybrid composite with ratio fiber percentage 25% glass fiber and 75% basalt fiber, improved the overall its flexural properties.

## 4. Conclusions

The mechanical properties of long basalt/woven-glass-fiber-reinforced unsaturated polyester-resin hybrid composites was investigated. The density of B composites with the addition of basalt fiber was slightly better at 1.8%, than B22.5/G7.5 hybrid composites. The tensile and flexural properties increased with the proportion of basalt fibers, the tensile strength was increased at 69% for B composites in relations to G composites, then further increased by 26% in relations to B22.5/G7.5 hybrid composites. The flexural strength was increased by 15% for B22.5/G7.5 hybrid composites than B composites.

## Figures and Tables

**Table 1:** Basalt/glass fiber composition in hybrid composites.

No.	Composites	Sample Designation	Matrix (wt %)	Fiber 30 (wt %)	
				Glass Fiber (wt %)	Basalt Fiber (wt %)
1.	UPE resin	UP	100	0	0
2.	5 layers of woven GF	GF	70	30	0
3.	2 layers woven BF + 3 layer of GF	B7.5/G22.5	70	7.5	22.5
4.	3 layers woven GF + 3 layer of BF	B15/G15	70	15	15
5.	4 layers BF + 2 layers woven GF	B22.5/G7.5	70	22.5	7.5
6.	5 layers BF	B	70	0	30

**Table 2:** Tensile properties of basalt/glass reinforced hybrid composites.

Samples	Tensile Strength (Mpa)	Tensile Modulus (Mpa)	Elongation at Break (%)
UP	8.14 ± 1.23 ^a^	290.57 ± 39.81 ^a^	4.52 ± 0.97 ^b^
G	126.33 ± 16.43 ^b^	3802.17 ± 658.15 ^b^	6.17 ± 0.84 ^c^
B7.5/G22.5	147.69 ± 15.27 ^b,c^	5519.80 ± 369.03 ^c^	4.42 ± 0.31 ^b^
B15/G15	168.89 ± 11.62 ^c^	6503.50 ± 94.37 ^d^	3.66 ± 0.43 ^b^
B22.5/G7.5	269.85 ± 23.35 ^e^	7106.32 ± 106.09 ^e^	2.38 ± 0.17 ^a^
B	213.92 ± 5.31 ^d^	6620.11 ± 431.21 ^f^	1.51 ± 0.22 ^a^

Values with different letters in the same column are significantly different (*p* < 0.5).

**Table 3:** Flexural properties of B/G/UP hybrid composites.

Samples	Flexural Strength (MPa)	Flexural Modulus (MPa)
UP	102.02 ± 20.88 ^a^	5091.64 ± 58.36 ^a^
G	215.72 ± 56.80 ^b^	14,546.90 ± 3041.57 ^b^
B7.5/G22.5	514.87 ± 47.45 ^c^	22,051.71 ± 1235.56 ^c^
B15/G15	584.54 ± 16.14 ^c^	32,971.43 ± 2164.44 ^d^
B22.5/G7.5	946.46 ± 54.48 ^e^	44,890.05 ± 2425.19 ^e^
B	819.78 ± 13.03 ^d^	36,513.83 ± 1429.48 ^d^

Values with different letters in the same column are significantly different (*p* < 0.5).
